# Prognostication after cardiac arrest

**DOI:** 10.1186/s13054-018-2060-7

**Published:** 2018-06-05

**Authors:** Claudio Sandroni, Sonia D’Arrigo, Jerry P. Nolan

**Affiliations:** 1 Istituto Anestesiologia e Rianimazione Università Cattolica del Sacro Cuore, Fondazione Policlinico Universitario “Agostino Gemelli, Largo Francesco Vito 1, 00168 Rome, Italy; 2 School of Clinical Science, University of Bristol, Bristol, UK; 3 Department of Anaesthesia and Intensive Care Medicine, Royal United Hospital, Bath, UK

**Keywords:** Cardiac arrest, Coma, Prognosis, Hypoxic brain damage

## Abstract

Hypoxic–ischaemic brain injury (HIBI) is the main cause of death in patients who are comatose after resuscitation from cardiac arrest. A poor neurological outcome—defined as death from neurological cause, persistent vegetative state, or severe neurological disability—can be predicted in these patients by assessing the severity of HIBI. The most commonly used indicators of severe HIBI include bilateral absence of corneal and pupillary reflexes, bilateral absence of N_2_O waves of short-latency somatosensory evoked potentials, high blood concentrations of neuron specific enolase, unfavourable patterns on electroencephalogram, and signs of diffuse HIBI on computed tomography or magnetic resonance imaging of the brain. Current guidelines recommend performing prognostication no earlier than 72 h after return of spontaneous circulation in all comatose patients with an absent or extensor motor response to pain, after having excluded confounders such as residual sedation that may interfere with clinical examination. A multimodal approach combining multiple prognostication tests is recommended so that the risk of a falsely pessimistic prediction is minimised.

## Background

About 80% of patients who are admitted to an intensive care unit (ICU) after resuscitation from out-of-hospital cardiac arrest (OHCA) are comatose [1] and two thirds of them will die because of hypoxic–ischaemic brain injury (HIBI) [2, 3]. Severe HIBI causes delayed neuronal death [4–6] and diffuse brain oedema [7, 8]. However, only a minority of these deaths occur as a direct consequence of massive neuronal injury (i.e. from brain death) [9]. In fact, most deaths caused by HIBI result from withdrawal of life-sustaining treatment (WLST) following prognostication of a poor neurological outcome [10, 11].

To avoid premature WLST in patients with a chance of neurological recovery, the risk of a falsely pessimistic prediction should be kept to a minimum. In other words, when predicting a poor neurological outcome, the false positive rate (FPR) (i.e. the ratio between the number of patients with a falsely pessimistic prediction divided by the number of patients with good neurological outcome) of the index used should ideally be zero, or their specificity should be 100%. However, even the most robust neurological predictors are not 100% specific; for this reason, the current guidelines [12, 13] recommend using a combination of predictors. These may include clinical neurological examination, electrophysiological investigations (electroencephalogram (EEG) and short-latency somatosensory evoked potentials (SSEP)), serum biomarkers, and neuroimaging. The characteristics of these categories of predictors are discussed in this article.

The aims of the present review are to summarise the current knowledge on the prediction of neurological outcome in patients who are comatose after CA and to provide practical recommendations on how to perform an accurate neuroprognostication in these patients.

## What represents a poor neurological outcome?

The most commonly used measure for reporting neurological outcome after CA is represented by Cerebral Performance Categories (CPCs) [14]. CPC 1 corresponds to the best possible outcome (no or minor disabilities) while CPC 5 corresponds to death (Table 1). The CPC was adapted from the Glasgow Outcome Scale (GOS) for traumatic head injury. The GOS scores correspond to those of the CPCs in inverse order; that is, GOS 1 corresponds to CPC 5 and vice versa. Despite its simplicity and widespread use, the CPC has been criticised for being too focused on mental function and less informative about body functions, activity, and participation [15], which may explain the reported lack of agreement between the CPC and subjective quality of life measures [16]. Alternatives to the CPC include the modified Rankin Scale (mRS) [17], which includes a 7-point scale ranging from 0 (no symptoms) to 6 (death), and the extended GOS (GOSE) [18]. The GOSE categories range from 1 (death) to 8 (upper good recovery) and include important information such as independence at home and outside home, work capacity, social activities, and return to normal life. All of these scales have limitations and none has been specifically designed to describe the outcome after global HIBI.

**Table 1:** Cerebral Performance Categories (CPCs) and Glasgow Outcome Scale (GOS)

CPC	GOS	Disability	Conscious	Independent	Features
1	5	No, or minor	Yes	Yes	Able to work and lead a normal life. May have mild dysphasia, non-incapacitating hemiparesis, or minor cranial nerve abnormalities
2	4	Moderate	Yes	Yes	Able to travel by public transport and work in sheltered environment Independent in activities of daily life. May have hemiplegia, seizures, ataxia, dysarthria, or memory changes
3	3	Severe	Yes	No	Limited cognition, dementia, locked-in, minimally conscious. Usually in institution, but it may be looked after at home with exceptional family effort
4	2	Unconscious	No	No	Persistent vegetative state
5	1	Dead	–	–	Certified brain dead or dead by traditional criteria

**Fig. 1:**
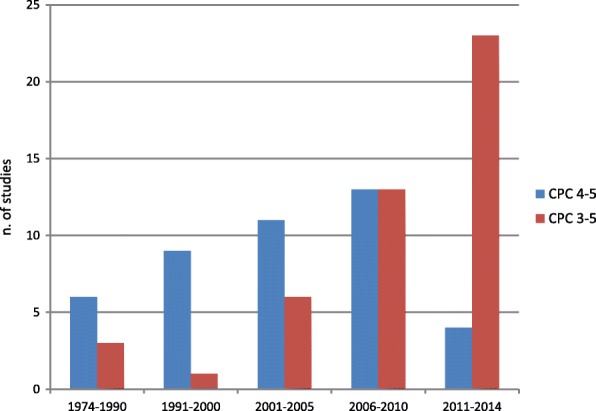
Definition of poor neurological outcomes in 87 prognostication studies, 1974–2014. Reproduced from [19], with permission. CPC Cerebral Performance Category

For clarity and for statistical purposes, in neuroprognostication studies the neurological outcome is generally dichotomised as ‘good’ or ‘poor’. However, there is no definite consensus on what represents a poor neurological outcome. Up to 2006, the majority of neuroprognostication studies defined poor outcome as CPC 4–5 (vegetative state or death) and a good outcome as CPC 1–3 (good neurological outcome and moderate to severe neurological disability). In the last 10 years, however, most studies included severe neurological disability (CPC 3) among the poor outcomes [19] (Fig. 1). This reflects different values and preferences in relation to neurological status after CA. These include giving priority to recovery of consciousness vs recovery of physical and neurological ability, and societal participation. Unfortunately, this heterogeneity causes confusion in the interpretation of results of neuroprognostication studies and prevents pooling the overall evidence in meta-analysis. For this reason, reporting the prevalence of all individual outcome categories in neuroprognostication studies would be desirable.

The latest version of the Utstein guidelines on outcome reporting after OHCA suggested that when dichotomising neurological outcome the CPC 3–5 threshold (or mRS 4–6) should be used for defining poor outcome. This definition will probably be updated if other outcome measures such as the GOSE are adopted to describe the outcome of CA in the near future.

The timing of neurological outcome assessment also affects its measured values, since in initially comatose cardiac arrest survivors neurological status can improve for up to 6 months after the event [20]. Optimal times for assessment of neurological outcome after cardiac arrest have yet to be established. However, 3 months after cardiac arrest seems to balance opportunity for observing improvement while minimising loss to follow-up [21].

### Health-related quality of life

Neurological status is a major determinant of overall functional outcome. However, measures of neurological status do not directly reflect overall functional outcome after cardiac arrest. Restoring the pre-arrest health-related quality of life (HRQOL) is the ultimate goal of resuscitation. Unfortunately, cardiac arrest survivors report cognitive impairment, restricted mobility, depression, and restricted societal participation after hospital discharge [22]. The recent ILCOR Advisory Statement on Core Outcome Set for Cardiac Arrest (COSCA) [23] in adults recommends including HRQOL assessed at a minimum of 3 months among the core outcome measures to be measured after cardiac arrest. Inclusion of HRQOL among measured outcomes in future neuroprognostication studies is desirable.

## Predictors of poor neurological outcome

### Clinical examination

**Fig. 2:**
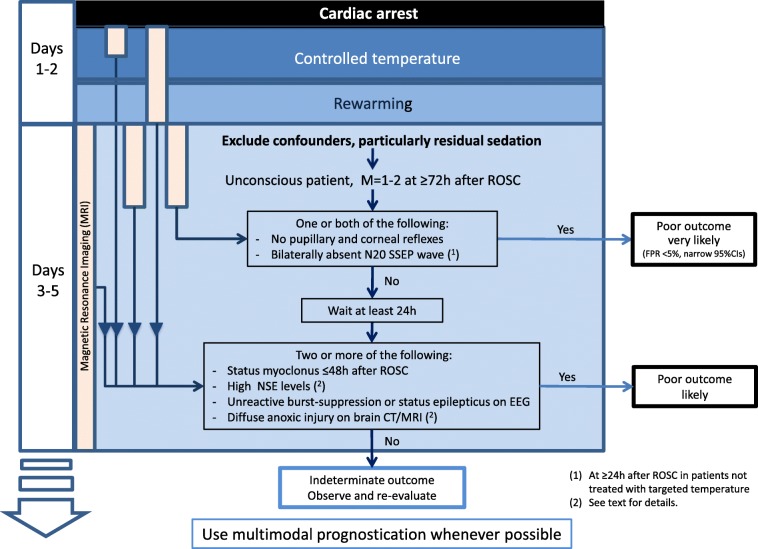
European Resuscitation Council–European Society of Intensive Care Medicine algorithm for neuroprognostication after cardiac arrest. Reproduced from [12] with permission. CI confidence interval, CT computed tomography, EEG electroencephalogram, FPR false-positive rate, M Glasgow Coma Scale Motor score, NSE neuron specific enolase, ROSC return of spontaneous circulation, SSEP short-latency somatosensory evoked potentials

A daily clinical neurological examination remains the foundation for prognostication [24]. The 2015 joint guidelines of the European Resuscitation Council (ERC) and the European Society of Intensive Care Medicine (ESICM) [12, 13] state that neuroprognostication can be considered in patients who, after having excluded major confounders such as residual sedation, are still unconscious and have an absent or extensor motor response to pain (Glasgow Coma Scale (GCS) Motor score ≤ 2) at 72 h or later after ROSC (Fig. 2). As a sign of poor neurological outcome, a GCS Motor score ≤ 2 at 72 h has low specificity but its sensitivity is high—around 70–80% [25, 26]—and it can therefore be used to identify patients with the most severe HIBI needing neuroprognostication.

A bilaterally absent pupillary light reflex (PLR) at ≥ 72 h from ROSC has a high specificity for predicting poor neurological outcome (FPR < 5% with narrow confidence intervals) [27]. However, its sensitivity is low [26, 28]. Moreover, standard PLR is a qualitative measure based on subjective assessment, which has raised some concerns about its reproducibility [29]. Automated infrared pupillometry provides a quantitative measure of pupil size, PLR, and constriction velocity, and is emerging as a novel modality to evaluate brainstem function at the bedside in critically ill patients [30]. A recent study in 103 post-CA comatose patients [31] showed that absence of pupillary reactivity measured with automated infrared pupillometry at 48 h after ROSC had higher specificity (100 (95% confidence interval 93–100)% vs 96 (86–99.5)%) and sensitivity (61 (48–75)% vs 43 (29–58)%) than standard PLR measured by certified neurologists. A multicentre prospective study (ClinicalTrials.gov NCT02607878) aiming to validate these results has been completed recently.

A bilaterally absent corneal reflex at 72 h after ROSC also indicates a likely poor outcome in patients who are resuscitated from CA. However, the specificity of the corneal reflex is slightly lower than that of the pupillary reflex (4 (1–7)% in seven studies in TTM-treated patients [12]). One reason for this could be that the corneal reflex is more prone to interference from residual effects of sedatives or muscle relaxants than PLR. Like PLR, the corneal reflex also has a low sensitivity.

#### Myoclonus

Myoclonus is a clinical phenomenon consisting of sudden, brief, involuntary jerks caused by muscular contractions or inhibitions. Presence of an early (≤ 48 h) post-anoxic status myoclonus—defined as a continuous and generalised myoclonus persisting for ≥ 30 min in a patient who is comatose after CA—is almost invariably associated with poor neurological outcome. In rare cases, however, an early-onset and generalised myoclonus may be associated with neurological recovery in these patients. Myoclonus is considered to be a less robust predictor than PLR and its use is recommended only in combination with other indices [13]. In particular, an EEG recording is recommended in order to rule out other more benign forms of post-anoxic myoclonus, such as Lance–Adams syndrome (LAS) [32, 33]. LAS is a post-anoxic action myoclonus, most often caused by asphyxial cardiac arrest, which becomes evident after awakening when a patient intentionally moves his/her limbs and is restricted to the limb being moved [33]. Elmer et al. [34] recently described two distinct EEG patterns in 65 patients with post-anoxic myoclonus: (a) a burst-suppression background with high-amplitude polyspikes in lock-step with myoclonic jerks; and (b) a continuous background with narrow, vertex spike-wave discharges in lock-step with myoclonic jerks. All patients with pattern (a) had poor outcome while 50% of patients with pattern (b) survived with LAS.

#### Limitations of clinical examination

As clinical examination is prone to interference from body temperature and from residual effects of sedatives and/or neuromuscular blocking drugs, these confounders should be carefully ruled out before starting the prognostication process. Another limitation of predictors based on clinical examination is that they cannot be concealed from the treating team and therefore their results may potentially influence clinical management and cause a self-fulfilling prophecy.

### Electrophysiology

#### Electroencephalogram

The EEG has long been used to assess the severity of HIBI [35]. However, its widespread adoption as a predictor has been hampered by the lack of a consistent classification of the different EEG patterns associated with poor neurological outcome [26]. Because of this inconsistency, the ERC–ESICM 2015 guidelines suggest considering malignant EEG patterns (status epilepticus or burst suppression after rewarming over an unreactive background) only in association with other predictors. A malignant EEG pattern not yet incorporated into the major guidelines is the suppressed background, defined as all EEG activity < 10 mV [36]. In comatose patients with HIBI, a substantial inter-rater agreement (κ = 0.71) among blinded assessors has been found [37] for the recognition of both burst suppression and suppressed background (with or without periodic discharges) defined according to the standardised terminology of the American Clinical Neurophysiology Society (ACNS) [36]. In a recent study on 103 resuscitated comatose patients [38], the presence of any of these two patterns on EEG recorded at a median of 77 h after ROSC predicted poor neurological outcome with 100 (88–100)% specificity and 50 (39–61)% sensitivity.

There is recent evidence showing that the EEG can provide important prognostic information even when it is recorded within the first 24 h after ROSC. In a study of 430 comatose resuscitated patients, poor neurological outcome (CPC 3–5) at 6 months was predicted accurately (specificity 100 (98–100)%) by one of the following patterns on continuous EEG: isoelectric, low-voltage (< 20 μV), or burst suppression with identical bursts [39]. However, the overall sensitivity of these signs was low (29 (22–36)%).

Another reason for monitoring the EEG in post-anoxic coma is to detect seizures, which may potentially cause secondary brain injury after HIBI. However, the benefit of aggressive treatment of post-anoxic seizures is still uncertain. A randomised trial (TELSTAR, ClinicalTrials.gov NCT02056236) is ongoing to answer this question.

#### Automated EEG analysis

The interpretation of EEG patterns in comatose survivors of CA is usually performed by neurophysiologists, and the assessment of continuous EEG requires the analysis of a considerable amount of data. Amplitude-integrated electroencephalography (aEEG) provides a simplified and, therefore, more suitable method for monitoring the EEG. In a study of 130 comatose resuscitated patients treated with targeted temperature management (TTM), absence of recovery to a continuous normal voltage within 36 h from ROSC on aEEG was 100 (93.5–100)% specific for poor neurological outcome at 6 months [40].

The bispectral index (BIS), an automated analysis of the EEG signal designed to monitor the depth of anaesthesia, has also been evaluated as a prognostic tool after CA. BIS values range from 100 (awake patient) to 0 (flat EEG). In two studies [41, 42], a BIS value of 6 or less during TTM, corresponding to a flat or low-amplitude EEG, predicted a poor neurological outcome with 0 (0–6)% FPR.

On a continuous EEG recording, persistence of a malignant pattern over time may be more significant than a single value. In a recent study where the BIS was recorded in 77 patients [43] a total duration of BIS 0 for 30.3 min predicted a poor neurological outcome with 63% sensitivity and 100% specificity (AUC 0.861; *p* = 0.007).

#### Short-latency somatosensory evoked potentials

Bilateral absence of the N_2_O cortical wave of SSEP at 72 h from ROSC predicts a poor neurological outcome with high accuracy and precision (FPR 0.4 (0–2)%) [25]. The ERC–ESICM guidelines include an absence of the N_2_O SSEP wave among the most robust predictors to be tested at 72 h after ROSC (Fig. 2). However, SSEP sensitivity rarely exceeds 50%. In other words, many patients destined to a poor neurological outcome after CA have a bilaterally present N_2_O SSEP wave. However, in these patients, lower N_2_O amplitudes are sometimes observed. Endisch et al. [44] measured the amplitude of the N_2_O SSEP waves between day 1 and day 4 after ROSC in 293 comatose CA survivors. An amplitude ≤ 0.62 μV had 100 (98–100)% specificity and 57 (48–65)% sensitivity for predicting a poor neurological outcome, defined as CPC 4–5. If an absence of the N_2_O SSEP wave had been adopted as a criterion for a positive test result, the SSEP sensitivity would have been 30%.

An advantage of SSEP over EEG is that they are less affected by sedation. However, they may be prone to electrical interference. In a large prospective prognostication study in comatose survivors of CA [45], the SSEP of three patients with good outcome were initially classified as being bilaterally absent during TTM, but post-hoc assessment from blinded neurophysiologists indicated that these three SSEP recordings were actually undeterminable because of excessive noise. In another 13 patients, SSEP were present during TTM but subsequently disappeared after rewarming. Current guidelines recommend recording SSEP only after rewarming.

### Biomarkers

Neuron specific enolase (NSE) and S-100B are protein biomarkers released following injury to neurons and glial cells, respectively. The rationale for their use for neuroprognostication is that their blood values are presumed to correlate with the extent of HIBI from CA [46]. Unlike clinical examination and EEG, concentrations of biomarkers are unlikely to be affected by sedatives and are easy to assess blindly, therefore preventing the self-fulfilling prophecy bias. However, biomarker blood values are continuous variables, which implies identifying a threshold when dealing with dichotomous outcomes such as the neurological prognosis of CA. Unfortunately, it is difficult to identify with a high degree of certainty a consistent biomarker threshold for identifying patients destined for a poor outcome. Biomarker thresholds vary with timing of measurement, reflecting their kinetics following initial release. An additional cause of inconsistency is the variability of techniques used to measure biomarkers, which can cause a significant systematic error between techniques [47]. For these reasons, unlike previous recommendations, [48] current guidelines [13] do not recommend any particular biomarker threshold to predict poor outcome with 100% specificity. An additional caveat for use of biomarkers is represented by extracerebral sources, which may cause false positive results. For NSE these include red blood cells, neuroendocrine tumours, and small cell carcinoma.

NSE is the most widely available and best documented biomarker of cerebral injury. In the largest study so far conducted on comatose survivors of CA (686 TTM-treated patients, 1823 samples assessed blind) [49], the NSE values corresponding to a false positive rate < 5% with the upper boundary of the 95% confidence interval within 5% were 61, 46, and 35 ng/ml at 24, 48 and 72 h from ROSC, respectively. Their corresponding sensitivities were 24, 59, and 63%. Serial measurement did not significantly improve the accuracy of prediction [50] over a single measurement at 48 h. However, sampling at multiple time points (24, 48, and 72 h) is recommended by current guidelines, in order to assess reproducibility and reduce the risk of a false positive result.

Another promising biomarker is tau protein, a marker of axonal injury. In a spin-off study of the TTM trial [51] the blood values of tau protein at 24, 48, and 72 h were measured using monoclonal antibodies in 689 patients. Results showed that a tau protein threshold of 11.2 ng/L at 72 h had 98 (96–99)% specificity and 66 (60–71)% sensitivity to predict poor neurological outcome (CPC 3–5) at 6 months. The area under the receiver operating characteristic (ROC) curve of tau protein at 72 h was higher than that of NSE (0.91 vs 0.86; *p* < 0.001). Its use, however, is still limited to specialised laboratories.

Recently, microRNAs (miRNAs) have been identified as candidate biomarkers for outcome prediction after CA. miRNAs are RNA molecules 20–22 nucleotides long which regulate gene expression. After global brain ischaemia, neuronal miRNAs cross the disrupted blood–brain barrier and can be measured in plasma. Their potential advantage is their ability to provide information not only on the severity of brain damage, but also on neuronal cell function. Preliminary studies [52] indicate that miR-124-3p is an independent predictor of both survival and neurological outcome in patients who are comatose after CA. Further investigation will be needed to confirm the clinical utility of miRNAs in HIBI.

### Near-infrared spectroscopy

Altered cerebral blood flow is considered one of the mechanisms causing HIBI [53].

Near-infrared spectroscopy (NIRS) is a non-invasive technique for monitoring regional cerebral oxygen saturation (SctO_2_) at the microvascular level. In a study of 107 comatose resuscitated patients [54], the mean SctO_2_ during the first 48 h after ROSC in patients with poor neurological outcome was significantly lower than in those with good neurological outcome at 6 months (66 ± 5% vs 68 ± 4%, respectively). Accuracy of SctO_2_, however, was low. At the best SctO_2_ threshold (55%), the sensitivity and specificity were 52% and 55% respectively and the area under the ROC curve was 0.58. Further studies will be needed to assess the usefulness of NIRS as a predictor of neurological outcome after CA.

### Imaging

#### Brain CT

The main CT finding of HIBI following CA is cerebral oedema, which appears as an attenuation of the grey matter/white matter (GM/WM) interface. This has been measured as the ratio (GWR) between the GM and the WM densities, which are usually sampled at three levels: basal ganglia, centrum semiovale, and high convexity. These changes occur early after CA. On brain CT performed in comatose survivors of CA between 1 and 24 h from ROSC, a GWR ranging between 1.16 and 1.22 predicted a poor neurological outcome (CPC 3–5) with 0% FPR and sensitivities ranging from 28 to 76% [55–59]. However, in a single-centre study including 240 patients with brain CT performed within 24 h from ROSC [60] a GWR < 1.22 predicted hospital mortality with high specificity (98 (91–100)%) but was unable to further characterise survivors between those having poor vs good outcome. The observed variability in GWR thresholds among studies may be due partly to the heterogeneity of the methods used for GWR calculation, while the variability in sensitivities may reflect the heterogeneous causes of arrest. Cerebral oedema is more common after arrest from non-cardiac causes [61].

There is presently no consensus on the optimal technique for measuring GWR nor on timing for performing brain CT for neuroprognostication in CA patients, although in the vast majority of studies the ROSC-to-CT interval was less than 24 h. A recent study [62] based on the TTM trial cohort showed that generalised oedema on brain CT detected visually by local radiologists without formal GWR measurement predicted poor neurological outcome (CPC 3–5) with 97.6 (91.8–9.4)% specificity and 14.4 (9.4–21.4)% sensitivity within 24 h from ROSC. The same findings from 24 h to 7 days after ROSC increased the specificity and sensitivity to 100 (87.9–100.0)% and 56.5 (47.3–65.3)% respectively.

#### Magnetic resonance imaging

HIBI after CA appears on brain MRI as hyperintense areas on diffusion weighted imaging (DWI). DWI changes are due to a reduction in the random motion of water protons, caused by a failure of the energy-dependent active water transport mechanisms due to HIBI. These changes can be quantified using the apparent diffusion coefficient (ADC). ADC thresholds for prediction of poor neurological outcome after CA have been measured as the whole-brain ADC [55, 63], the proportion of brain volume with low ADC [64, 65], and the lowest ADC value in specific brain areas that are most commonly affected by HIBI [66]. These include the occipital cortex, deep grey nuclei, hippocampus, and cerebellum. MRI was very accurate for predicting poor neurological outcome in individual studies, but the methods used to calculate the severity of the ischaemic lesions in the brain are heterogeneous.

Current prognostication guidelines suggest performing brain MRI 2–5 days after ROSC. This timing is based on results of early studies [67]; however, recent evidence [55, 68] showed that MRI can predict neurological outcome as early as 3 h after ROSC.

Given the few patients studied, the spatial and temporal variability of post-anoxic changes in both CT and MRI, and the lack of standardisation for quantitative measures of these changes, current guidelines suggest using brain imaging studies for prognostication after CA only in combination with other predictors and in centres where specific experience is available.

MRI has limited feasibility in the most unstable patients, and this may also have introduced a selection bias in prognostic studies based on MRI.

## Predictors of good neurological outcome

The vast majority of evidence on neuroprognostication after cardiac arrest concerns prediction of poor neurological outcome. However, some predictors of good neurological outcome have been identified in recent years. Although these have not yet been included in international guidelines, they may indicate the potential for recovery in patients with uncertain prognosis and reduce the risk of an inappropriate WLST. Most of these predictors are based on electrophysiology and include the presence of a continuous or nearly continuous EEG within 12 h from ROSC [69], presence of early EEG reactivity [69, 70], and improvement of auditory discrimination (an analysis of EEG responses to auditory stimuli) from the first to the second day after ROSC [71]. Absence of DWI abnormalities on MRI within 1 week of ROSC is also highly suggestive of good neurological outcome [67].

## Suggested prognostication strategy

Most TTM-treated patients recover consciousness within 72 h from ROSC [72]. The ERC–ESICM guidelines on post-resuscitation care [13] recommend the neuroprognostication algorithm reported in Fig. 2 for all patients who remain comatose with an absent or extensor motor response to pain at ≥ 72 h from ROSC. Results of earlier prognostic tests should also be considered at this time. Before prognostic assessment is performed, major confounders must be excluded; these may include sedation, neuromuscular blockade, hypothermia, severe hypotension, and metabolic or respiratory derangements.

The most robust predictors (FPR < 5% for prediction of poor outcome with narrow confidence interval documented in > 5 studies from at least three different groups of investigators) should be evaluated first. These include bilaterally absent pupillary reflexes at ≥ 72 h after ROSC and/or a bilaterally absent N_2_O SSEP wave after rewarming. If none of these signs is present, less robust predictors with wider confidence intervals and/or an inconsistent definition or threshold are considered. These include the presence of early (< 48 h) status myoclonus, high serum NSE values at 48–72 h after ROSC, an unreactive malignant EEG pattern (burst suppression, status epilepticus) after rewarming, and the presence of diffuse ischaemic injury on brain CT within 24 h after ROSC or on brain MRI at 2–5 days after ROSC. Combining at least two of these predictors is suggested.

If none of these criteria is present or if the results from prognostic tests are discordant, the prognosis is indeterminate and prolonged observation and treatment is continued so that late awakeners can be identified. In 15–30% of patients with an eventually good outcome, awakening may occur between 48 h and 10–12 days after discontinuing sedation [72, 73]. Patients with renal insufficiency, older age, or post-resuscitation shock have an increased risk of delayed awakening [72]. The presence of predictors of neurological recovery (see earlier) should also be considered in this context. In patients with prolonged unconsciousness (2–4 weeks after ROSC), advanced MRI techniques—whole-brain white matter fractional anisotropy (WWM-FA) measured using diffusion tensor imaging [74]—may predict poor neurological outcome more accurately than conventional MRI.

## Multimodality

Almost all prognostication studies have a low or very low quality of evidence, the main reason being the risk of self-fulfilling prophecy (SFP). This bias occurs when the treating team is not blinded to the results of the prognostic index under investigation and use it to decide on WLST. Among 73 studies included in a review published in 2014 [12], only nine (12%)—three of which were from the same group—addressed SFP by blinding, and only 37 (51%) reported the criteria for WLST. Given the relatively small sample size of most prognostication studies and the risk of SFP, even the most robust predictors cannot predict outcome with absolute certainty, and for this reason a multimodal approach is recommended. The algorithm suggested in the current ERC–ESICM guidelines [13] is per se multimodal, since it adds the results of clinical examination to those of electrophysiology, biomarkers, or imaging. However, this approach is based on expert opinion. Future prospective studies will be needed to confirm whether this model is able to increase the precision of specificity without greatly reducing sensitivity.

## Conclusions

Patients who are comatose at 72 h or more after ROSC and in whom major confounders have been excluded should undergo prognostication, aimed to detect signs of severe and irreversible HIBI. This can be achieved using four main categories of tests: clinical examination, electrophysiology, biomarkers, and neuroimaging. The timing of these tests varies and may precede the clinical assessment at ≥ 72 h that initiates the prognostication process. Among prognostic tests, ocular reflexes and somatosensory evoked potentials are considered the most robust, while biomarkers, electroencephalography, imaging, and status myoclonus have inconsistencies which suggest using them only in combination. A multimodal approach combining multiple prognostication tests is recommended by current guidelines so that the risk of a falsely pessimistic prediction is minimised.

## Abbreviations

ADC: Apparent diffusion coefficient
AUC: Area under the receiver operating characteristic curve
BIS: Bispectral index
CA: Cardiac arrest
CPC: Cerebral Performance Category
CT: Computer tomography
EEG: Electroencephalogram
ERC: European Resuscitation Council
ESICM: European Society of Intensive Care Medicine
FPR: False-positive rate
GOS: Glasgow Outcome Scale
GWR: Grey matter/white matter ratio
HIBI: Hypoxic–ischaemic brain injury
LAS: Lance–Adams syndrome
MRI: Magnetic resonance imaging
mRS: Modified Rankin Scale
NIRS: Near-infrared spectroscopy
NSE: Neuron specific enolase
OHCA: Out-of-hospital cardiac arrest
PLR: Pupillary light reflex
ROSC: Return of spontaneous circulation
SFP: Self-fulfilling prophecy
SSEP: Short-latency somatosensory evoked potentials
TTM: Targeted temperature management
WLST: Withdrawal of life-sustaining treatment
